# Effects of chiropractic spinal manipulation on laser-evoked pain and brain activity

**DOI:** 10.1186/s12576-021-00804-2

**Published:** 2021-06-24

**Authors:** Benjamin Provencher, Stéphane Northon, Carlos Gevers Montoro, Julie O’Shaughnessy, Mathieu Piché

**Affiliations:** 1 Department of Anatomy, Université du Québec à Trois-Rivières, 3351 boul. des Forges, C.P. 500, Trois-Rivières, QC G9A 5H7 Canada; 2 CogNAC Research Group, Université du Québec à Trois-Rivières, Trois-Rivières, QC G9A 5H7 Canada; 3 Madrid College of Chiropractic, Madrid, Spain; 4 Department of Chiropractic, Université du Québec à Trois-Rivières, Trois-Rivières, QC G9A 5H7 Canada

**Keywords:** Spinal manipulation, Hypoalgesia, Nociceptive fibers, Electroencephalography

## Abstract

The aim of this study was to examine the mechanisms underlying hypoalgesia induced by spinal manipulation (SM). Eighty-two healthy volunteers were assigned to one of the four intervention groups: no intervention, SM at T4 (homosegmental to pain), SM at T8 (heterosegmental to pain) or light mechanical stimulus at T4 (placebo). Eighty laser stimuli were applied on back skin at T4 to evoke pain and brain activity related to Aδ- and C-fibers activation. The intervention was performed after 40 stimuli. Laser pain was decreased by SM at T4 (*p* = 0.028) but not T8 (*p* = 0.13), compared with placebo. However, brain activity related to Aδ-fibers activation was not significantly modulated (all *p* > 0.05), while C-fiber activity could not be measured reliably. This indicates that SM produces segmental hypoalgesia through inhibition of nociceptive processes that are independent of Aδ fibers. It remains to be clarified whether the effect is mediated by the inhibition of C-fiber activity.

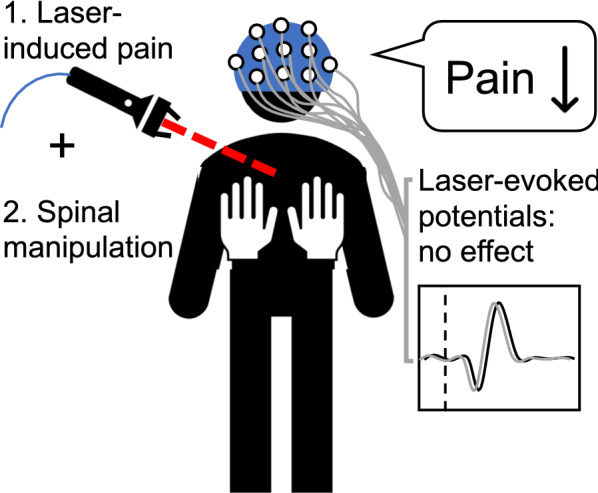

## Background

Low back pain and neck pain are among the leading causes of years lived with disability [35]. Recent clinical practice guidelines for these spinal disorders recommend self-management as well as physical and psychological therapies as first-line treatment, while pharmacotherapy and surgery are recommended when first-line treatments were ineffective [13, 21, 37, 58, 62, 63]. Currently, spinal manipulative therapy (SMT) is recommended for the management of spine pain by most of these clinical practice guidelines [12, 13, 21, 37, 58, 62]. However, the neurophysiological mechanisms underlying the pain-relieving effects of SMT are still unclear. The understanding of these mechanisms could improve clinical practice by providing optimal conditions in which SMT is most likely to provide pain relief.

The mechanisms by which SMT may relieve pain have been examined in previous studies and reviewed recently [4, 27]. One potential mechanism is the inhibition of neural processes underlying temporal summation of pain (TSP), the perceptual correlate of wind-up [29]. Wind-up is an increased excitability of dorsal horn neurons caused by repetitive stimulation of afferent C-fibers [29]. It is thought to share common mechanisms with central sensitization and hyperalgesia [1, 29], making it a relevant process for the investigation of pain relief by SMT. Decreased TSP on the leg (lumbar dermatome) was reported following lumbar SMT [25]. These results were later replicated and were shown to be specific to spinal manipulation (SM) in healthy volunteers and patients with low back pain (LBP) [6–8]. In contrast, no reduction in TSP was observed between sham and SMT in patients with LBP [2]. However, procedures used in this study were different from previous studies so discrepancies may be explained by methodological differences. In line with the inhibition of C-fiber-related pain by SM, it was shown that TSP produced by repeated electrical stimulation in the back is inhibited by SM, while pain produced by a single electrical pulse is not [60].

Altogether, these studies suggest that SM inhibits nociceptive processes related to C-fiber activation, while those related to Aδ-fiber activation are unaffected. However, this remains to be examined with neurophysiological methods that allow selective activation of nociceptive fibers and measurement of their activity, since behavioral methods alone are not sufficient for this purpose.

The aim of this study was to determine how brain activity associated with the activation of nociceptive fibers is modulated by SM. Based on the behavioral results of previous studies [6–8, 25, 60], we hypothesized that pain and pain-related brain activity related to C but not A-δ fibers would be inhibited by SM.

## Methods

### Experimental design

This study relied on a mixed design to compare changes in laser-evoked pain and brain activity between four groups. A random-number generator was used to create a randomization sequence and assign participants to one of the four experimental groups: no intervention (*n* = 20), placebo intervention (light mechanical stimulus segmental to laser-evoked pain; *n* = 21), SM segmental to laser-evoked pain (SM-T4: *n* = 21) and SM heterosegmental to laser-evoked pain (SM-T8: *n* = 20).

Choosing a placebo intervention for spinal manipulation is challenging. No placebo intervention can account for all aspects of SM [57]. A commonly used placebo intervention consists of a skin contact with no thrust, or soft pressing [57]. The intervention aims at reproducing the spinal manipulation set up and the contact with the participant. In the present study, we selected this intervention as placebo intervention and skin contact was achieved with a hand-held dynamometer to standardize the applied force. This procedure is identical to that used in a previous study [60]. In addition to the placebo group, we included a control group to measure non-specific temporal effects, in which no intervention was applied.

### Participants

**Fig. 1:**
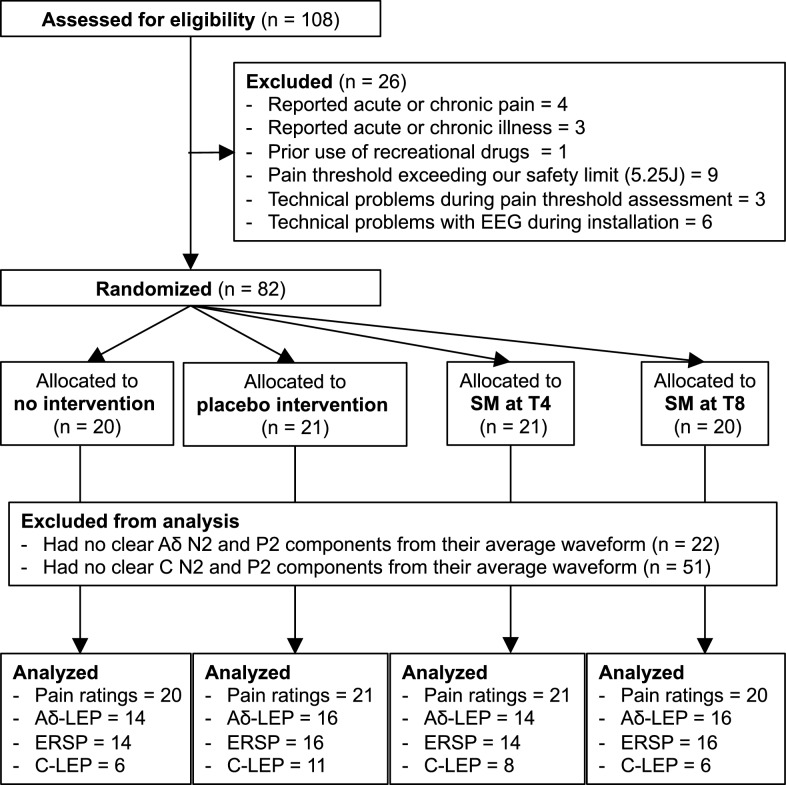
Flow diagram of enrollment, allocation, and analysis. *SM* spinal manipulation; *LEP* laser-evoked potential, *ERSP* event-related spectral perturbations

A flowchart detailing the participants inclusion in the study and analyses is presented in Fig. 1. Eighty-two healthy volunteers (40 men and 42 women; aged 26.6 ± 7.8 years [mean ± SD]) were recruited by advertisement on the campus of Université du Québec à Trois-Rivières. Participants were included if they were between 18 and 55 years old. They were excluded if they reported acute or chronic pain, acute or chronic illness, psychiatric disorders, if they underwent spinal surgery, or took any medication or recreational drugs during the 2 weeks prior to experimentation.

### Experimental protocol

**Fig. 2:**
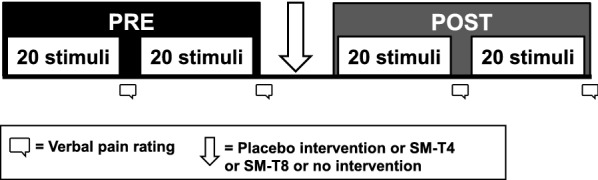
Experimental paradigm. Participants received four blocks of 20 laser stimuli for a total of 80 stimuli. After each block, participants were instructed to rate pain verbally. Between block 2 and 3, participants received either no intervention, the placebo intervention, spinal manipulation at T4 (SM-T4) or spinal manipulation at T8 (SM-T8). Laser-evoked pain and brain activity were averaged over 40 stimuli and were compared before and after the intervention (PRE vs POST conditions)

During the experiment, room temperature was kept constant at 24 °C while participants comfortably lay prone on a chiropractic table. Their head was slightly elevated by a folded towel placed under their chin to avoid putting pressure on the electrooculography (EOG) and frontal electrodes. The participant and experimenter wore safety glasses designed for a 1340-nm wavelength laser. Participants were instructed to keep their eyes open, look at a fixation cross to minimize eye movement and refrain movement as much as possible during stimulation. The experiment comprised 80 laser stimuli delivered with an inter-stimulus interval that varied between 8 and 10 s. After each set of 20 stimuli, participants provided pain ratings and could blink as needed during a pause of 60 s. The intervention (SM or placebo) was performed after 40 stimuli, which were used as baseline for data analyses (see PRE condition in Fig. 2).

### Laser stimulation

Painful stimuli were produced by laser heat pulses generated by an infrared neodymium-doped yttrium aluminum perovskite laser (Nd:YAP, DEKA 1340; Electronical Engineering, Florence, Italy). These stimuli have been shown to activate nociceptors selectively [11, 55]. The stimulation protocol was identical to the protocol used in a previous study reporting an increased ability to detect C-fiber laser-evoked potentials [31], except for the target (back instead of hand dorsum) and the smaller number of stimuli. The laser beam was transmitted through a 10-m optic fiber cable. Laser pulse duration was set to 5 ms and laser beam to 7 mm (≈38.5 mm^2^ area). Based on safety recommendations for repeated laser stimuli [40], a maximum fluence limit was set to 14 J/cm^2^ (5.25 J intensity limit for a 7 mm diameter). The laser was triggered using a stimulus presentation software (Spike2; Cambridge Electronic Design Limited, Cambridge, UK). To avoid stimulation of the same area more than once per block, 25 ink marks were drawn on the area to be stimulated in the back with a regular Hi-Tecpoint 0.5-mm Black Pilot pen, in a 5 × 5 cm grid centered around T4 spinous process (T4–T5 dermatome). The laser stimuli were targeting the marks, but the diameter of the laser beam far exceeded the size of the marks. The laser was moved to the next point of the grid after each stimulus. This procedure is safe for experimentation using Nd:YAP laser [40].

Individual pain threshold was determined using a staircase procedure. Before pain threshold assessment, participants were instructed to focus on the warm/burning sensation in their back and to report pain intensity verbally after each stimulus using a numerical rating scale ranging from 0 to 100, 0 indicating “no pain” and 100 “the worst pain imaginable”. Stimuli were delivered at an initial intensity of 0.5 J and stimulus intensity increased sequentially by 0.5 J increments until pain was reported (rating of 1/100 or higher), or until the 5.25 J safety limit was reached. If no pain was reported (rating of 0/100) at the highest energy laser stimulus within our safety limits (5.25 J), the participant was excluded from the study. This is necessary for the purpose of the study, in which we examine pain inhibition and not only LEPs. Nine participants were excluded for this reason. Otherwise, the energy was increased sequentially again until a pain rating of at least 30/100 was reported or until the 5.25 J limit was reached. Participants were then familiarized with the selected intensity using five consecutive stimuli with an inter-stimulus interval varying between 5 and 10 s. If the intensity was deemed acceptable for the participant, the experiment was continued. If the participant judged that the stimulus intensity produced pain that could not be tolerated for the duration of the experiment, stimulus intensity was decreased by 0.5 J and the familiarization procedure resumed until an acceptable stimulus intensity was reached. Once approved by the participant, the stimulus intensity was kept constant throughout the experiment.

### Spinal manipulation and placebo intervention

A previous study has shown that hypoalgesic effects can be produced by a SM at T4, where pain was applied (segmental SM) [60]. In the present study, T4 and T8 segments were selected for segmental and heterosegmental SM, for this reason and for practical reasons; with spinal manipulation at these segments, the participant can lie down comfortably without moving for the intervention. This allows artifact-free recording of brain activity. SM were performed by a licensed chiropractor and consisted in a short-duration, high-velocity, low-amplitude, posterior-to-anterior thrust applied with both hands. SM was applied over the transverse processes of T4 or T8 vertebrae to generate audible release (cavitation). This type of manipulation lasts less than 200 ms and involves a force of approximately 500 N [30, 64]. Immediately after receiving SM, participants were asked to report if the intervention was painful. No pain was reported in 95% (39/41) of participants. In two participants, light pain was reported only for the short thrust phase. The placebo intervention consisted of a 20-N force applied for 2 s over the spinous process of T4 using a hand-held dynamometer (Hoggan scientific LLC, model Micro FET2, Salt Lake City, UT, USA).

It is not possible to make participants blind to the intervention in the case of spinal manipulation. However, participants were not informed about where the intervention (SM or placebo) was delivered, that other participants would receive different interventions and what was the effect expected in this experiment. No adverse event was reported by participants following SM or the placebo intervention.

### Pain ratings

After each series of 20 stimuli, participants were instructed to rate pain verbally using a numerical rating scale ranging from 0 to 100, 0 indicating “no pain” and 100 “worse pain imaginable”. They were instructed to report the average pain induced by the 20 stimuli.

### Expectations of pain modulation

Expectations of pain modulation were measured using a visual analogue scale [26]. Before the experiment, participants were presented a form with the following question: “On the scale below, indicate the change in laser-pain intensity that you expect following the intervention in your back”. The scale was a horizontal line ranging from -100 to 100 with the following anchors: -100 = “maximum decrease”, 0 = “no change” and 100 = “maximum increase”.

### Electroencephalographic recordings

Electroencephalography (EEG) was recorded using a 64-channel BrainVision system with active Ag–AgCl electrodes mounted on an actiCAP according to the International 10–20 system (Brain Products, Gilching, Germany). Electrodes were nose-referenced, and the ground was set at FPz. Signals were sampled at 1000 Hz and filtered using a 0.01–100 Hz band-pass filter. Eye movements and blinks were recorded using right eye EOG with electrodes placed at the suborbital ridge and just lateral to the external ocular canthus. Electrodes impedance was kept below 20 kΩ.

### Laser-evoked potentials (LEP) analysis

EEG signals were analyzed offline using EEGLAB v.13.5.4b [19]. After applying a 0.5–30 Hz finite impulse response (FIR) band-pass filter and re-referencing to the common average, data were segmented into epochs extending from − 100 ms to + 1500 ms relative to stimulus onset. Epochs were baseline corrected using the − 100 to 0 ms window and then visually inspected to reject those most likely containing artifacts (amplitude value exceeding ± 100 µV). On average 3.2 ± 2.4 out of 80 epochs (4%) were rejected. After, an Infomax-independent component analysis (ICA) was applied using the in-built EEGLAB function Runica to identify and remove components associated with noise (e.g., eye movement, eye blinks, cardiac, and muscle artifacts).

Finally, average waveforms were computed for each participant and condition. LEP components of interest, including the N2 and P2 [31, 36, 51, 61] were extracted from these waveforms. From the 82 participants tested, 22 (26.8%) did not have clear Aδ-N2 and Aδ-P2 peaks and 51 (62.2%) did not have clear C-N2 and C-P2 peaks from their average waveforms. This was assessed independently by three of the experimenters (BP, SN and MP). The N2 and P2 calculations were thus performed on data from the remaining 60 participants for Aδ-fiber LEP and on data from the remaining 31 participants for C-fiber LEP (see Fig. 1 for the distribution of these participants among experimental groups). The Aδ-N2 was defined as the first major negative deflection occurring between 170 and 400 ms with a maximum amplitude at the vertex (Cz), and the Aδ-P2 was defined as the first major positive deflection occurring between 280 and 500 ms with a maximum amplitude at the vertex (Cz). The C-N2 was defined as the first major negative deflection occurring between 450 and 600 ms with a maximum amplitude at the vertex (Cz) that followed the Aδ-P2, and the C-P2 was defined as the first major positive deflection occurring between 550 and 800 ms with a maximum amplitude at the vertex (Cz) that followed the C-N2. Latencies vary depending on stimulus location. For the back, the latency of A-delta fibers LEP remains the same regardless of the spinal level stimulated (from C5 to L5) [16, 22, 34]. However, the latencies of C-fibers LEP vary depending on the distance between the stimulus location and the brain [34, 59]. The latencies reported for C-fibers LEP in the present study are consistent with previous findings for stimuli applied to the upper back (T2, T4, T6, T8) [34, 59]. Besides, peak amplitude was calculated for each component instead of peak-to-peak, since each peak originates from different brain generators and reflects distinct neural processes [24, 43, 46].

### Event-related spectral perturbations analysis

Event-related spectral perturbations (ERSP) [52] were examined for two reasons. Firstly, since nociceptive C-fibers have a largely variable response latency, averaging multiple trials to obtain laser-evoked potentials reduces amplitude [31, 39]. Accordingly, time-domain analyses only give partial access to the evoked brain activity [45]. Therefore, LEP and ERSP analyses are complementary and allow the measurement of brain responses that are phase-locked to stimulus onset or not. ERSP were used in previous studies investigating C-fibers laser-evoked brain activity [20, 31, 36, 44]. Secondly, we were interested in measuring gamma-band oscillations for their potential as a reliable biomarker of nociception and pain [28, 56].

ERSP were analyzed with EEGLAB. After applying a 1–100 Hz FIR band-pass filter and re-referencing to the common average, data were segmented into epochs extending from -2000 ms to + 2000 ms relative to stimulus onset. Epochs were baseline corrected using the − 700–− 200 ms window and, as described above, visual inspection and ICA were applied to remove artifacts. A Morlet wavelet convolution [45] was computed using the channel time–frequency options available in EEGLAB v.13.5.4b [19]. Two hundred time points were generated, and 100 linearly spaced frequencies were computed from 1 to 100 Hz. Variable cycles were used for low and high frequencies, with 3 cycles for lowest frequencies and up to 15 cycles for highest frequencies [19]. This variable number of cycles allows for the wavelet convolution method to provide a better frequency resolution at lower frequencies and a better temporal resolution at higher frequencies. ERSP data were computed in decibels relative to baseline for all electrodes separately. The time–frequency data of all trials were averaged for each participant and condition separately, resulting in 2 average time–frequency maps for each electrode.

From these maps, the mean power was extracted from the Cz electrode in the following regions of interest (time × frequency) based on previous studies reviewed in [56]: from 2 to 10 Hz between 150 and 400 ms, from 8 to 29 Hz between 300 and 1000 ms, from 30 to 60 Hz between 100 and 350 ms, and from 61 to 100 Hz between 150 and 350 ms. The gamma-band was separated as low and high gamma based on previous studies [3, 15]. The ERSP values for each time–frequency point included in the regions of interest were extracted from each subject. A mean ERSP value was then obtained for each participant and regions of interest by selecting and averaging the values with the 20% highest or lowest amplitude (for power increase or decrease relative to baseline) [32, 33, 45, 48]. The mean power calculations were performed on data from the same 60 participants used for Aδ-LEP analysis.

### Statistical analysis

Statistical analysis was conducted using Statistica v13.1 (Kivuto Solutions Inc., Ottawa, ON, Canada). All results are expressed as mean ± SD. SD values were corrected to remove between-subject variability [14] and statistical threshold was set at p < 0.05. Data distribution was assessed for normality with the Kolmogorov–Smirnov test and homogeneity of variance was assessed using Levene’s test. Since both tests indicated that the assumptions for using analysis of variance (ANOVA) were met, pain intensity, Aδ-N2 and Aδ-P2 peak amplitude and latency, as well as ERSP values were analyzed using mixed ANOVA with one between-subject factor (groups, 4 levels) and one within-subject factor (time, 2 levels). Planned contrasts were used to decompose significant effects. For C-N2 and C-P2 analyses, the number of participants that could be included was limited and data was not normally distributed. Thus, groups were compared on C-N2 and C-P2 amplitude modulation using the Kruskal–Wallis H tests. Effect sizes are reported based on partial eta-squared (*η*^*2*^_*p*_).

## Results

### Pain intensity

**Fig. 3:**
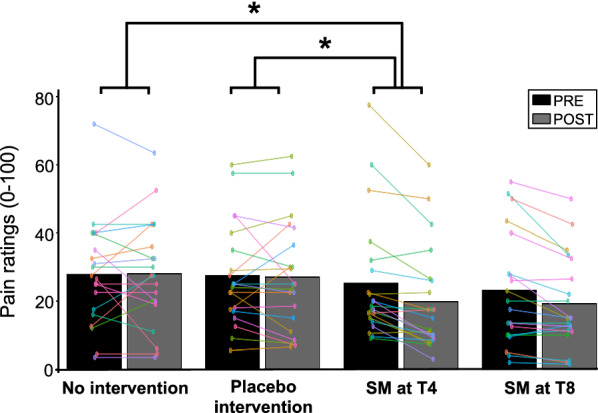
Pain modulation. Mean pain ratings before and after the intervention for the four groups. SM at T4 (segmental) produced pain inhibition compared with no intervention or the placebo intervention. SM at T8 produced similar effects but they were not statistically significant compared with no intervention or the placebo intervention. Data from each participant are represented by linked colored dots and the mean of these data points for each condition are represented by black and grey bars. *P < 0.05

The mean stimulus intensity for each group was 5.0 ± 0.4 for no intervention, 4.5 ± 1.3 for the placebo intervention, 4.8 ± 0.7 for SM-T4 and 4.7 ± 0.6 for SM-T8. No significant difference was observed between groups (F_3,78_ = 1.4, *p* = 0.24; *η*^*2*^_*p*_ = 0.05). Pain ratings are reported in Table 1 and presented in Fig. 3. Pain intensity was significantly different between groups over time (interaction: *F*_3,78_ = 2.97, *p* = 0.037, *η*^*2*^_*p*_ = 0.10). Planned contrasts revealed that pain intensity was significantly decreased by SM at T4 compared with no intervention (*p* = 0.013) and the placebo intervention (*p* = 0.028). In contrast, SM at T8 did not modulate pain significantly compared with no intervention (*p* = 0.07) and the placebo intervention (*p* = 0.13). Moreover, the placebo intervention did not modulate pain compared with no intervention (*p* = 0.74). This indicates that pain inhibition by SM at T4 (homosegmental to pain stimulation) was greater than changes produced by non-specific temporal effects (no intervention) and placebo effects (light mechanical stimulation), while SM at T8 (heterosegmental to pain stimulation) did not produce significant effects. The same results were obtained with the 60 participants included in the EEG analyses (see below).

**Table 1:** Pain ratings for the four experimental groups (mean ± SD)

	No intervention	Placebo intervention	SM at T4	SM at T8
PRE	27.8 ± 4.6	27.5 ± 3.9	25.2 ± 2.8	23.1 ± 2.6
POST	28.1 ± 4.6	27.0 ± 3.9	19.8 ± 2.8	19.2 ± 2.6

### Expectations of pain relief do not predict pain inhibition

Expectations of pain relief were 15.1 ± 27.8 for the placebo intervention, 20.2 ± 21.1 for SM at T4 and 13.8 ± 16.7 for SM at T8, with no significant difference between groups (*F*_2,59_ = 0.5, *p* = 0.6, *η*^*2*^_*p*_ = 0.02). To examine whether greater expectation of pain relief predicted pain inhibition, simple regression analyses were performed. Pain inhibition was not predicted by expectations for the placebo (*R*^2^ = 0.08, *p* = 0.22), SM at T4 (*R*^2^ = 0.16, *p* = 0.07) or SM at T8 (*R*^2^ = 0.005, *p* = 0.76).

### Laser-evoked potentials

**Fig. 4:**
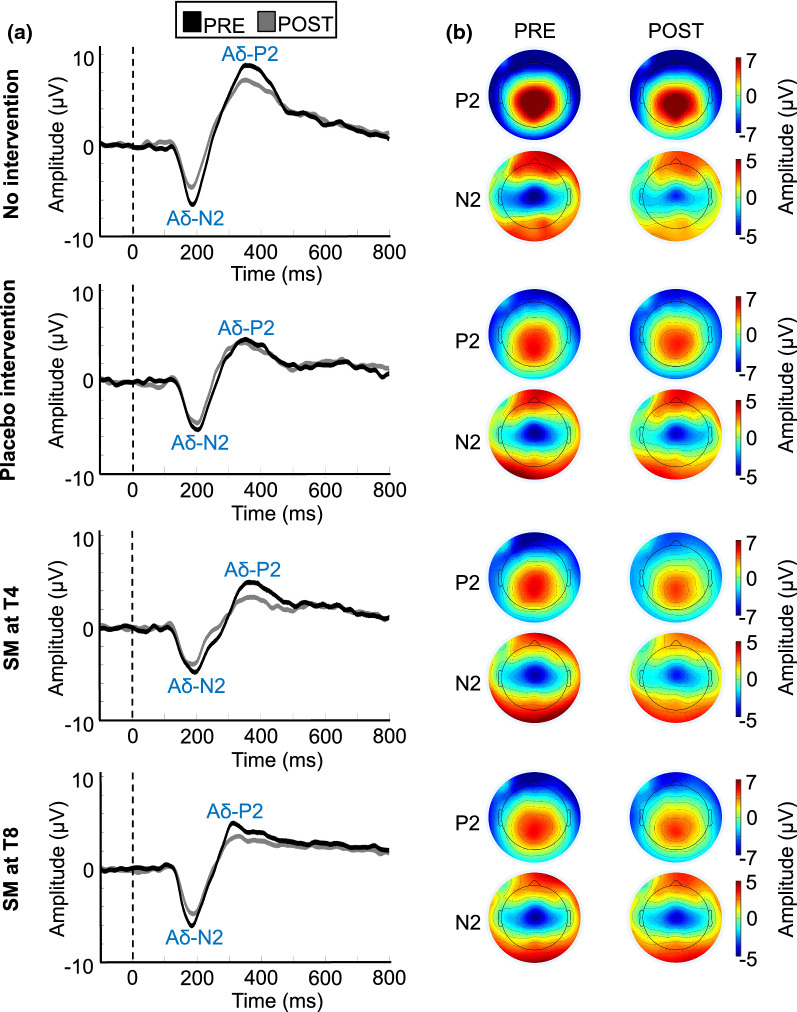
Aδ-fiber laser-evoked potentials.** a** Average waveforms time-locked to the onset of laser stimulation for the N2 and P2 at Cz with a nose reference, for the 60 participants included in the analysis. **b** Average topographic maps for the Aδ-N2 (200 ms) and Aδ-P2 (365 ms). No significant effect was observed between groups

**Fig. 5:**
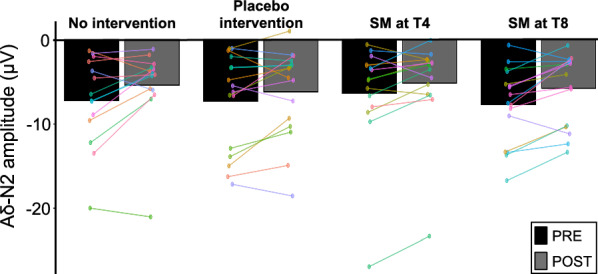
Aδ-P2 amplitude. Mean P2 peak amplitude. No significant effect was observed between groups. Data from each participant are represented by linked colored dots and the mean of these data points for each condition are represented by black and grey bars

**Fig. 6:**
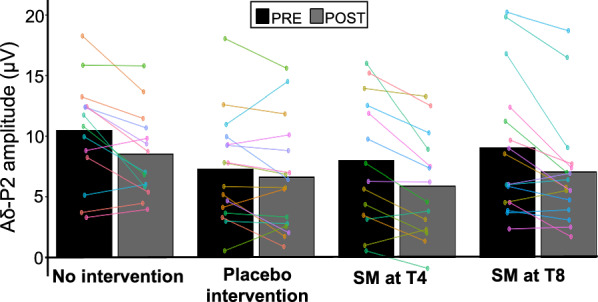
Aδ-N2 amplitude. Mean **A**δ-N2 peak amplitude. No significant effect was observed between groups. Data from each participant are represented by linked colored dots and the mean of these data points for each condition are represented by black and grey bars

**Fig. 7:**
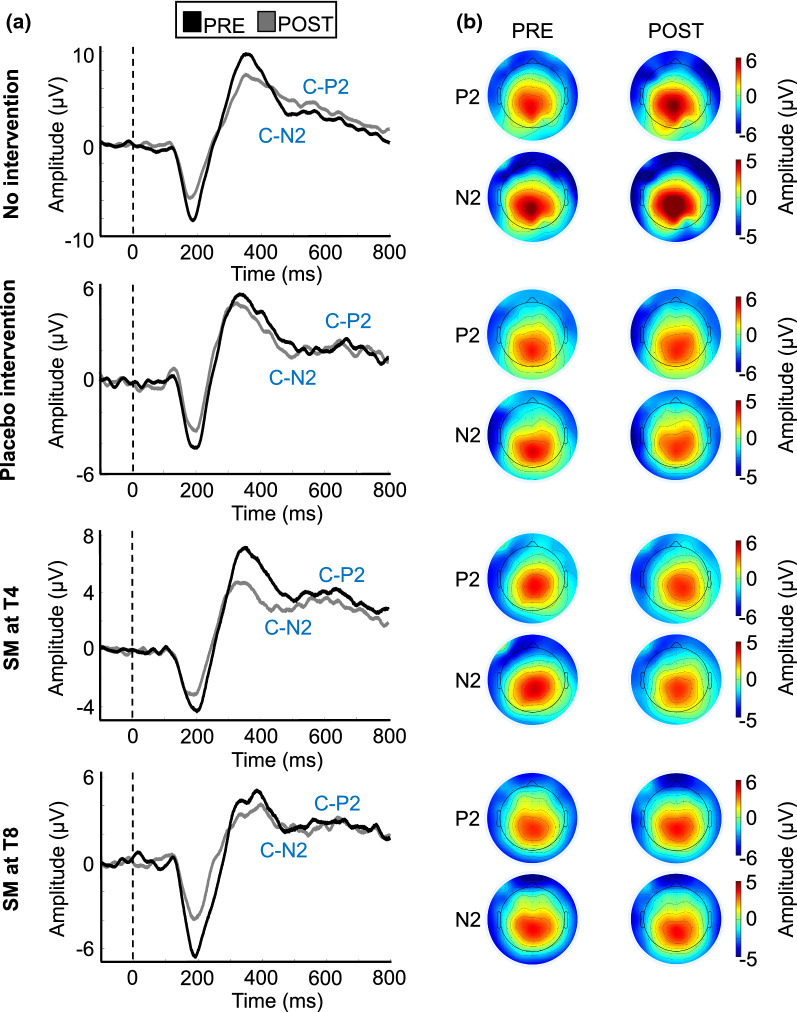
C-fiber-evoked potentials.** a** Average waveforms time-locked to the onset of laser stimulation for the N2 and P2 at Cz with a nose reference, for the 31 participants included in the analysis. **b** Average topographic maps for the C-N2 (500 ms) and C-P2 (650 ms). No significant effect was observed between groups

Average waveforms and topographic maps for the N2 and P2 components are presented in Figs. 4, 5, 6 and 7. As expected, both components show a central scalp distribution and are maximal at the vertex.

#### Aδ-*N2 and* Aδ-P2 *peak amplitude and latency*

Aδ-N2 peak amplitudes and latencies are presented in Tables 2 and 3 and Fig. 5. Aδ-N2 peak amplitude decreased over time (main effect: F_1,56_ = 24.49, p < 0.001, *η*^*2*^_*p*_ = 0.30), but this effect was not significantly different between groups (interaction: F_3,56_ = 0.45, p = 0.72, *η*^*2*^_*p*_ = 0.02). Aδ-N2 peak latency was not significantly different between groups (main effect: F_3,56_ = 0.78, p = 0.51, *η*^*2*^_*p*_ = 0.04) or between groups over time (interaction: F_3,56_ = 0.98, p = 0.41, *η*^*2*^_*p*_ = 0.05).

Aδ-P2 peak amplitudes and latencies are presented in Tables 2 and 3 and Fig. 6. Aδ-P2 peak amplitude decreased over time (main effect: F_1,56_ = 34.1, p < 0.001, *η*^*2*^_*p*_ = 0.38), but this effect was not significantly different between groups (interaction: F_3,56_ = 1.52, p = 0.22, *η*^*2*^_*p*_ = 0.08). Aδ-P2 peak latency was not significantly different between groups (main effect: F_3,56_ = 1.0, p = 0.40, *η*^*2*^_*p*_ = 0.05) or between groups over time (interaction: F_3,56_ = 0.36, p = 0.78, *η*^*2*^_*p*_ = 0.02).

**Table 2:** N2 and P2 peak amplitude (µV) for the four experimental groups (mean ± SD)

			No intervention	Placebo intervention	SM at T4	SM at T8
Aδ	N2	PRE	− 7.2 ± 1.5	− 7.3 ± 1.2	− 6.4 ± 1.0	− 7.7 ± 1.1
		POST	− 5.4 ± 1.5	− 6.2 ± 1.2	− 5.1 ± 1.0	− 5.7 ± 1.1
	P2	PRE	10.5 ± 1.1	7.3 ± 1.0	8.0 ± 1.1	9.0 ± 1.2
		POST	8.5 ± 1.1	6.6 ± 1.0	5.9 ± 1.1	7.0 ± 1.2
C	N2	PRE	2.2 ± 0.71	0.49 ± 0.46	1.8 ± 0.78	0.42 ± 1.3
		POST	3.5 ± 0.71	0.25 ± 0.46	1.0 ± 0.78	0.57 ± 1.3
	P2	PRE	4.4 ± 0.82	3.8 ± 0.42	5.4 ± 0.62	3.8 ± 1.2
		POST	4.9 ± 0.82	3.5 ± 0.42	4.8 ± 0.62	4.2 ± 1.2

**Table 3:** N2 and P2 peak latency (ms) for the four experimental groups (mean ± SD)

			No intervention	Placebo intervention	SM at T4	SM at T8
Aδ	N2	PRE	201.9 ± 8.5	220.8 ± 9.1	217.0 ± 7.5	224.1 ± 15.3
		POST	195.6 ± 8.5	208.4 ± 9.1	214.9 ± 7.5	223.6 ± 15.3
	P2	PRE	377.9 ± 21.4	398.3 ± 41.7	407.4 ± 35.8	398.8 ± 16.2
		POST	361.0 ± 21.4	393.0 ± 41.7	411.5 ± 35.8	401.8 ± 16.2
C	N2	PRE	543.7 ± 13.3	513.8 ± 11.0	476.6 ± 16.5	483.0 ± 9.4
		POST	544.7 ± 13.3	521.2 ± 22.0	465.7 ± 16.5	489.2 ± 9.4
	P2	PRE	603.5 ± 17.0	601.7 ± 16.5	577.8 ± 21.0	596.4 ± 8.1
		POST	594.9 ± 17.0	609.9 ± 16.5	575.8 ± 21.0	586.6 ± 8.1

#### C-N2 and C-P2 peak amplitude and latency

**Fig. 8:**
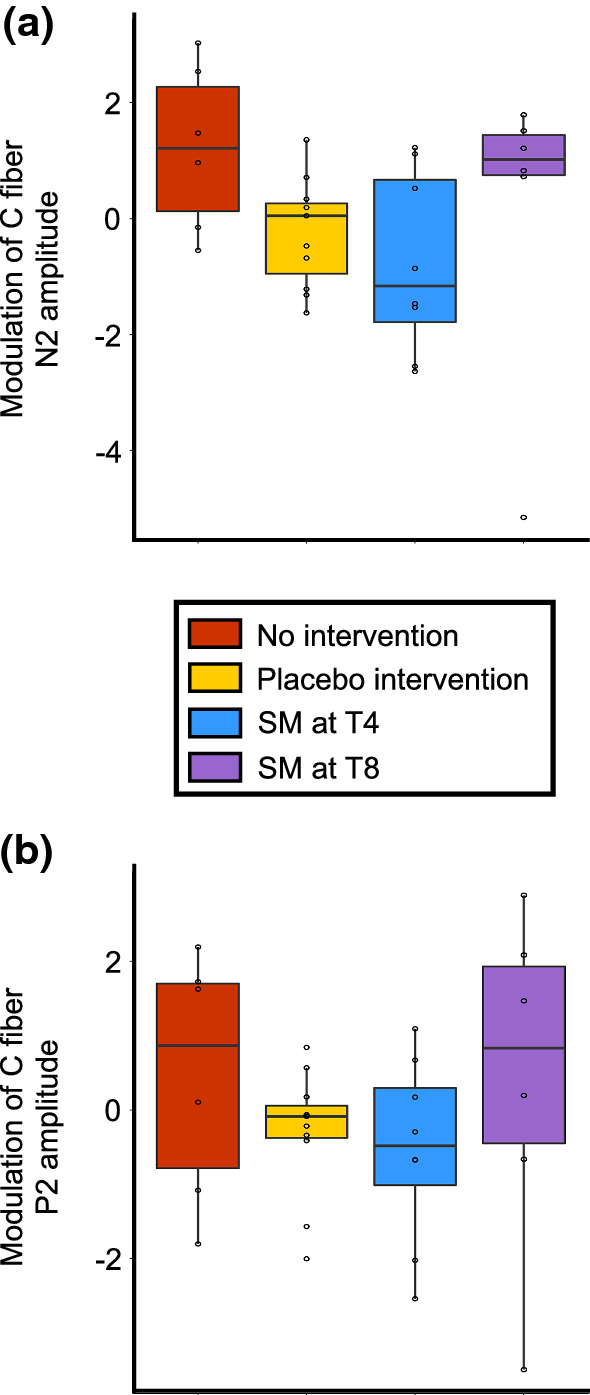
Modulation of C-fiber-evoked potentials. **a** Boxplot of the difference in C-fibers N2 amplitude. **b** Boxplot of the difference in C-fibers P2 amplitude. PRE values were subtracted from POST values. A negative score reflects inhibition. No significant effect was observed between groups. Data from each participant are represented by black circles

C-P2 peak amplitudes and latencies are reported in Tables 2 and 3 and presented in Fig. 8. Due to the low number of participants included in these analyses and low statistical power, no conclusion could be reached, and the results are presented as pilot data only. The Kruskal–Wallis H test revealed no significant difference between groups for the C-N2 peak (H(3) = 7.3, p = 0.062), the C-P2 peak (H(3) = 3.0, p = 0.40), the C-N2 latency (H(3) = 1.7, p = 0.63), and C-P2 latency (H(3) = 1.9, p = 0.59).

### Event-related spectral perturbations

**Fig. 9:**
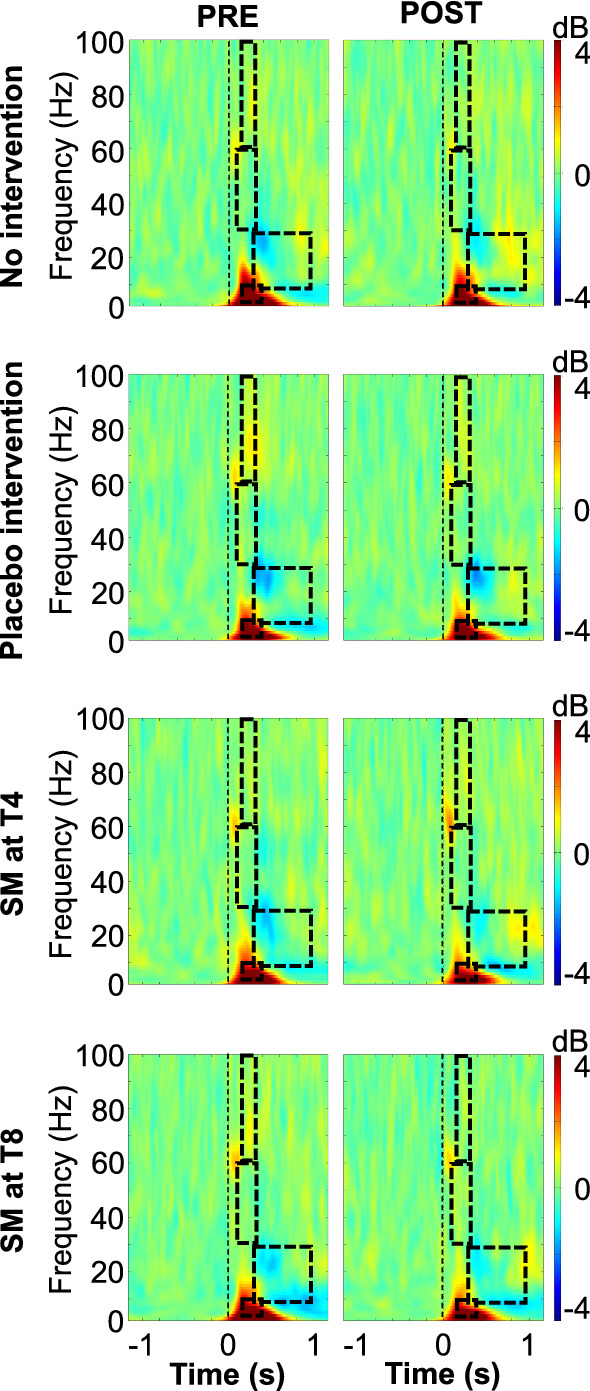
Time–frequency analyses**.** Average time–frequency maps, time-locked to the onset of laser stimulation. Units are in decibels relative to baseline (− 700–− 200 ms). Regions of interest are highlighted by the dashed rectangles. No significant effect was observed between groups

Averaged ERSPs and regions of interest are presented in Fig. 9.

#### 2–10 Hz

**Fig. 10:**
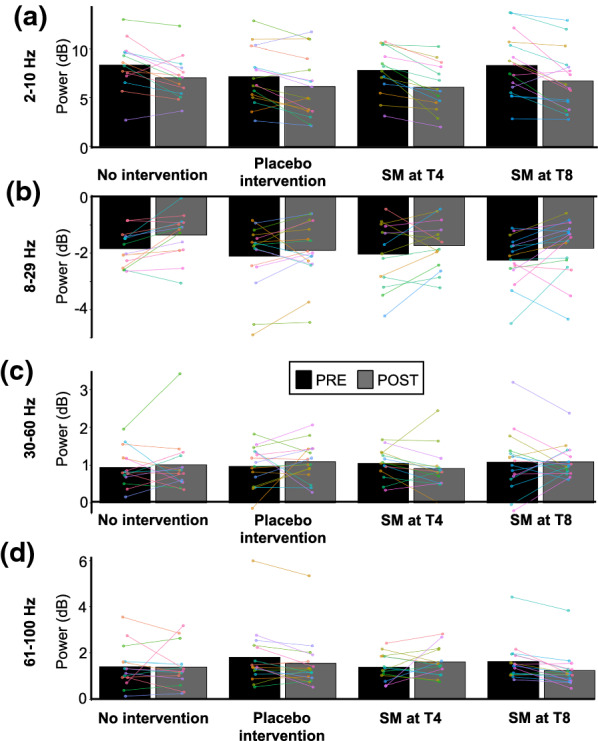
Quantitative time–frequency analyses. Mean ERSP values from the four regions of interest. **a** from 2 to 10 Hz between 150 and 400 ms, **b** from 8 to 29 Hz between 300 and 1000 ms, **c** from 30 to 60 Hz between 100 and 350 ms, **d** from 61 to 100 Hz between 150 and 350 ms. No significant effect was observed between groups. Data from each participant are represented by linked colored dots and the mean of these data points for each condition are represented by black and grey bars

The mean power in the 2–10 Hz region of interest is presented in Table 4 and Fig. 10a. It decreased over time (main effect: *F*_1,56_ = 52.7, *p* < 0.001, *η*^*2*^_*p*_ = 0.48), but this effect was not significantly different between groups (interaction: *F*_3,56_ = 0.68, *p* = 0.57, *η*^*2*^_*p*_ = 0.04).

**Table 4:** ERSP power (dB) for the four experimental groups (mean ± SD)

		No intervention	Placebo intervention	SM at T4	SM at T8
2–10 Hz	PRE	8.4 ± 0.8	7.2 ± 0.7	7.8 ± 0.7	8.3 ± 0.9
	POST	7.0 ± 0.8	6.1 ± 0.7	6.1 ± 0.7	6.7 ± 0.9
8–29 Hz	PRE	− 1.8 ± 0.3	− 2.1 ± 0.3	− 2.0 ± 0.4	− 2.2 ± 0.4
	POST	− 1.4 ± 0.3	− 1.9 ± 0.3	− 1.7 ± 0.4	− 1.8 ± 0.4
30–60 Hz	PRE	0.9 ± 0.3	1.0 ± 0.3	1.1 ± 0.2	1.1 ± 0.3
	POST	1.0 ± 0.3	1.1 ± 0.3	0.9 ± 0.2	1.1 ± 0.3
61–100 Hz	PRE	1.4 ± 0.4	1.8 ± 0.2	1.4 ± 0.4	1.6 ± 0.2
	POST	1.4 ± 0.4	1.6 ± 0.2	1.6 ± 0.4	1.3 ± 0.2

#### 8–29 Hz

The mean power in the 8–29 Hz region of interest is presented in Table 4 and Fig. 10b. It increased over time (main effect: *F*_1,56_ = 13.5, *p* < 0.001, *η*^*2*^_*p*_ = 0.19), but this effect was not significantly different between groups (interaction: F_3,56_ = 0.45, p = 0.72, *η*^*2*^_*p*_ = 0.02).

#### 30–60 Hz

The mean power in the 30–60 Hz region of interest is presented in Table 4 and Fig. 10c. It was not significantly different over time (main effect: *F*_1,56_ = 0.05, *p* = 0.82, *η*^*2*^_*p*_ = 0.0009) or between groups over time (interaction: *F*_3,56_ = 0.55, *p* = 0.65, *η*^*2*^_*p*_ = 0.03).

#### 61–100 Hz

The mean power in the 61–100 Hz region of interest is presented in Table 4 and Fig. 10d. It was not significantly different over time (main effect: *F*_1,56_ = 1.70, *p* = 0.20, *η*^*2*^_*p*_ = 0.03) or between groups over time (interaction: *F*_3,56_ = 2.62, *p* = 0.06, *η*^*2*^_*p*_ = 0.12).

## Discussion

The aim of this study was to determine the neurophysiological mechanisms of SM-induced hypoalgesia by examining changes in nociceptive brain activity evoked by laser stimulation. The expected decrease in nociceptive brain activity would reflect a downstream inhibition of spinal nociceptive transmission considering that SM-induced hypoalgesia is presumed to be caused by spinal processes [7, 8, 25, 50]. The present results indicate that SM applied segmentally, but not heterosegmentally to the laser stimulus reduces laser-evoked pain compared with a placebo intervention and non-specific temporal effects. Brain activity related to Aδ fiber activation were not significantly modulated by the intervention, confirming our hypothesis that SM-induced hypoalgesia is produced by inhibition of nociceptive processes that are independent of Aδ fibers. However, brain activity related to C-fibers could not be measured reliably so it remains to be clarified whether SM-induced hypoalgesia relies on the inhibition of activity related to C-fibers.

Pain inhibition by SM was examined in several studies using mechanical, electrical, chemical (capsaicin) or thermal stimuli [42]. Generally, these studies indicate that SM produces hypoalgesia. A series of studies also indicate that pain inhibition by SM relies more specifically on processes related to C-fiber activity [6, 7, 9, 25, 50]. This conclusion was based on the lack of SM-induced hypoalgesia when pain was evoked by a single contact heat stimulus, while TSP produced by a repetition of the same stimulus at a frequency known to produce wind-up was decreased. In most of these studies, painful stimuli were applied on the leg, in a lumbar dermatome related to vertebral segments where SM was applied and not on the back, to allow measuring specific effects on Aδ and C-fibers [25]. A subsequent study confirmed that inhibition of TSP by SM could also be observed when painful stimuli are applied on the back, at the site of SM [60]. In that study, pain evoked by a single electric pulse was not inhibited while pain evoked by a repeated application of the same stimulus (TSP) was decreased. In the present study, the lack of inhibition of Aδ-fibers’ activity was shown experimentally with measures of brain activity. This is the first study to investigate the neurophysiological effects of SM using laser stimulation, which allows the activation of nociceptive afferents selectively and the assessment of nociceptive brain activity [11, 55]. In addition to LEPs, ERSPs were examined. The lack of effect of SM on pain-related ERSPs is consistent with some of the findings in a previous study, in which the effects of SM on central processing of tonic pain were examined [47]. Indeed, no significant change was observed in delta, theta, alpha and beta frequency bands after one session of multiple SM in healthy volunteers. Furthermore, since laser stimuli activate Aδ and C-fibers, the significant pain inhibition observed in the SM-T4 group is likely caused by inhibition of C-fiber-related processes. However, this could not be confirmed because laser-evoked C-fiber activity could not be measured reliably in most participants, although the experimental protocol was adapted to allow detecting such activity [31]. It is possible that the number of stimuli was not sufficient to obtain optimal signal to noise ratio, but 40 stimuli per condition was a compromise to avoid tissue damage or non-specific temporal effects. It is also possible that the different innervation of back and hand skin by nociceptors makes C-fiber LEP measurement more difficult in the back. However, a recent study indicates that the spatial acuity for pain is comparable between these two regions [41] so it is unlikely that sparser innervation in the back explains the lack of measurable C-fiber LEP in the present study. Nevertheless, the shorter distance between the skin of T4 region and the brain may not allow Aδ- and C-fiber responses to be distinguished as clearly as for the hand. Therefore, it remains to be clarified whether C-fiber activity is inhibited by SM and whether this contributes to pain inhibition or whether SM has a specific effect on pain amplification processes such as wind-up and the resulting TSP. Future studies are needed to optimize stimulation protocols and experimental designs to examine the modulation of nociceptive brain activity by SM with laser stimulation in the back. Another issue to address is the non-specific attenuation of laser-evoked brain responses, including Aδ- and C-fiber responses. The amplitude of some nociceptive responses measures in the present study attenuated over time. It is known that stimulus saliency and their evoked neurophysiological responses decrease as the number of stimuli of constant intensity increases [33, 61]. However, it may be possible to limit this attenuation by delivering the laser stimuli among non-painful stimuli, making laser stimuli unpredictable, novel and more salient [17, 18, 38].

In the present study, we also examined whether nociceptive activity and laser-evoked pain may be modulated by a light mechanical stimulus (the placebo intervention). The lack of pain inhibition by this placebo intervention suggests that SM produced specific effects and that SM-induced hypoalgesia relies at least in part on the activation of deep high-threshold mechanoreceptors. This is congruent with current knowledge on SM hypoalgesic mechanisms. A body of knowledge from animal and human studies indicates that spinal manipulation transiently increases the discharge of type Ia, Ib and II afferents in paraspinal tissues [53, 54]. These afferents and the nociceptive afferents project to the dorsal horn of the spinal cord where they may interact [53]. Although this remains to be clarified, the inhibition of TSP by SM may represent a perceptual correlate of this spinal interaction [25, 60]. In addition, the present results corroborate findings from a previous study in which no significant hypoalgesia occurred following the application of a light mechanical stimulus [60]. Yet, Penza et al. reported a similar inhibition of TSP when comparing low-velocity, low-amplitude spinal mobilization to SMT [50]. Nevertheless, the amount of force applied during this mobilization was not measured, so it may have been sufficient to activate deep high-threshold mechanoreceptors.

Previous studies have examined how psychological factors may contribute to SM-induced hypoalgesia [5, 8, 10]. In the present study, to limit a potential bias induced by knowledge on SM, chiropractors or chiropractic students were not included in the recruited sample. Indeed, knowledge on SM and their pain-relieving effects may be associated with expectation of pain relief, which may affect the results. In addition, expectations of pain relief were measured, and this confirmed that expectations were not significantly different between groups. Moreover, expectations did not predict pain inhibition. Thus, it is unlikely that pain inhibition by SM was caused by expectations in the present study.

In the present experimental study, we included healthy volunteers with no spine disorder, no spine pain, and no clinical indication for SM. Several studies have demonstrated the hypoalgesic effects of SM performed on arbitrarily selected spinal segments in healthy volunteers [6, 9, 25, 50, 60]. This indicates that SM produces hypoalgesic effects even in the absence of a clinical indication. Whether the hypoalgesic effects measured in healthy volunteers are different compared with patients with back pain or spine disorders remains to be clarified [65].

Regarding heterosegmental effects, inhibition of laser-evoked pain was not significant when SM was performed at T8 (SM-T8 group). However, a similar trend as for the segmental SM (SM-T4 group) was observed so we cannot exclude that weaker, but significant effects may be observed with larger samples. Also, T4 and T8 vertebrae are relatively close to each other so this result suggests that the hypoalgesic effects may show a gradient, with the amplitude of the effects decreasing as a function of the distance from the manipulated segment. Previous studies reported a lack of pain inhibition by heterosegmental SM [7, 25, 50], but in these studies, the heterosegmental SM was in a different spinal region. Besides, although the SM technique used in the present study aims at specific joints (T4–T5), several joints are mobilized [23, 49].

## Conclusion

In summary, SM can reduce pain evoked by laser stimulation in the back and this hypoalgesic effect does not involve nociceptive processing related to Aδ-fiber activation. It remains to be confirmed whether C-fiber activity is inhibited by SM and whether this contributes to its pain-relieving effects.

## Data Availability

The dataset supporting the conclusions of this article is available from the corresponding author on reasonable request.

## Abbreviations

EEG: Electroencephalography
EOG: Electrooculography
ERSP: Event-related spectral perturbations
FIR: Finite impulse response
LBP: Low back pain
LEP: Laser-evoked potential
NdYAP: Neodymium-doped yttrium aluminum perovskite laser
SM-T4: Spinal manipulation at T4 (segmental to laser-evoked pain)
SM-T8: Spinal manipulation at T8 (heterosegmental to laser-evoked pain)
SM: Spinal manipulation
SMT: Spinal manipulative therapy
TSP: Temporal summation of pain
